# Supplementary material for: Cost of Illness Analysis of Type 2 Diabetes Mellitus: The Findings from a Lower-Middle Income Country
Source: Int J Environ Res Public Health. 2022 Oct 2;19(19):12611. doi: 10.3390/ijerph191912611 (PMC9566593; doi:10.3390/ijerph191912611)
Supplement: Supplementary file 1 [file ijerph-19-12611-s001.zip › ijerph-1901742-supplementary.pdf]

Questionnaire

Title: Cost of diabetes care in out-patient clinics.

ID No.				

Personal identifier sheet

1	Gender	2	Age Yrs.	DOB (m/d/y)	3	Marital status
4	How identified as Diabetic?	5	Total time taken in Interview		6	Date
7	Language of interview	8	Interviewer Name		9	Signature of Interviewer
	Urdu: ____________<br>Other (specify):<br>__________________					

Status
Complete: ____________
Incomplete: ____________

SUPERVISOR
Checked: ___________
Validated: __________

Socio-demographic and general information

ID No.				

Q #	Question	Code	Skip pattern	Response
Q # 1	What is your current status of education?	Primary …………. 1<br>Secondary ……..... 2<br>Intermediate ….….. 3<br>Graduate & above..4<br>Madresah ……..…. 5<br>Can read & write…6<br>Illiterate …………7<br>Other ……………. 0		
Q # 2	What is your marital status?	Single …………..…. 1<br>Married …………..…2<br>Other ……………..... 0	If 1, go to Q # 4	
Q # 3	How many alive children do you have?	Actual (in numbers)		
Q # 4	What is your employment status?	Office Job ...……. 1<br>Businessman …..... 2<br>Laborer ………… 3<br>Unemployed …… 4<br>Other ……..……... 0	If 4, Go to Q # 6	
Q # 5	What is your total monthly income?	Actual (in Rs.)	Now Go to Q # 8	
Q # 6	Reason for not being employed?	Jobless ……….......1<br>Student ……..……2<br>Housewife …..….. 3<br>Retired …………..4<br>Unable ……….….5<br>Other …. ….....…0		
Q # 7	If unemployed, what are your activities in most of the days of week?	Actual		
Q # 8	What is overall household income?	Actual (in Rs.)		
Q # 9	How many people are supported on this income?	Actual (in numbers)		
Q # 10	Do you own? (in your home)			
	(a) Television	Yes………………1<br>No ………………2		
	(b) Refrigerator	Yes………………1<br>No ………………2		
	(c) Air Conditioned	Yes………………1<br>No ………………2		
	(d) Motor-Cycle	Yes………………1<br>No ………………2		
	(e) Washing Machine	Yes………………1<br>No ………………2		
	(f) Computer	Yes………………1<br>No ………………2		
	(g) Car	Yes………………1<br>No ………………2		

Health status and behaviour information

ID No.				

Q #	Question	Code	Skip pattern	Response
Q # 11	Since how long you have been diagnosed as diabetic?	Actual (Years)		
Q # 12	How often you visit to your doctor due to diabetes?	(Per Year)		
Q # 13	Do you follow the doctor's advice for?			
	(a) Consultations	Yes……………. 1<br>No ……………. 2	If 1, Go to Q # 15	
	(b) Laboratory investigations	Yes……………. 1<br>No ……………. 2	If 1, Go to Q # 15	
	(c) Taking medicines	Yes……………. 1<br>No ……………. 2	If 1, Go to Q # 15	
	(c) Dietary Intake	Yes……………. 1<br>No ……………. 2	If 1, Go to Q # 15	
Q # 14	Why you do not follow Doctor's advice?	No knowledge …1<br>No resources ..... 2<br>No time ………. 3<br>Other …….….. 0		
Q # 15	How do you currently treat the diabetes?	Diet plan/Exercise ..1<br>Diabetes tablets . .. 2<br>Insulin . . . . 3<br>Combination . . . . . 4<br>Other 0		
Q # 16	Do you need someone to help in your diabetes care?	Yes ……………. 1<br>No …………….. 2		

Co-morbidities/complications & treatment information

ID No.				

Q #	Question	Code	Skip pattern	Response
Q # 17	Please tell us about the co-morbidities / complication due to diabetes. Do you have?			
	(a) Hypertension	Yes . . . . 1<br>No 2		
	(b) Dyslipidemia	Yes . . . . 1<br>No 2		
	(c) Depression	Yes . . . . 1<br>No 2		
	(d) Heart disease	Yes . . . . 1<br>No 2		
	(e) Retinopathy	Yes . . . . 1<br>No 2		
	(f) Neuropathy	Yes . . . . 1<br>No 2		
	(g) Nephropathy	Yes . . . . 1<br>No 2		
	(h) Other ________________	Yes . . . . 1<br>No 2		
Q # 18	Do you use medication for?			
	(a) Hypertension	Yes . . . . 1<br>No 2		
	(b) Dyslipidemia	Yes . . . . 1<br>No 2		
	(c) Depression	Yes . . . . 1<br>No 2		
	(d) Heart disease	Yes . . . . 1<br>No 2		
	(e) Retinopathy	Yes . . . . 1<br>No 2		
	(f) Neuropathy	Yes . . . . 1<br>No 2		
	(g) Nephropathy	Yes . . . . 1<br>No 2		
	(h) Other ________________	Yes . . . . 1<br>No 2		

Treatment regime information

ID No.				

Q #	Question	Dose	Frequency	Expenses
Q # 19	Which medicines you use for diabetes and other co-morbidities / complications in last month?			
	(a)			
	(b)			
	(c)			
	(d)			
	(e)			
	(f)			
	(g)			
	(h)			
	(i)			
	(j)			
	(k)			
	(l)			
	(m)			
	(n)			
Q # 20	Do you use insulin to treat your diabetes?	Yes . . . . 1<br>No 2	If 2, go to Q # 22	
Q # 21	Use of insulin?	**Dose**	**Frequency**	**Expenses**

__Diabetes cost information__

ID No.				

Q #	Question	Code	Skip pattern	Response
Q # 22	How much money you spent for?	In current visit (In rupees)		
	(a) consultation	Actual		
	(b) lab tests	Actual		
	(c) medicines	Actual		
	(d) travel cost	Actual		
	(e) food cost	Actual		
	(f) other health care cost	Actual		
Q # 23	How much time you spent during?	In current visit (in minutes)		
	(a) Travelling to clinic	Actual		
	(b) Waiting in clinic	Actual		
	(c) Consultation	Actual		
Q # 24	For how long you take leave from your employer for each visit?	0 hours …..….. 1<br>1-2 hours ……. 2<br>2-4 hours ……. 3<br>1 day ..……….. 4<br>Other 0		
Q # 25	Does anyone accompany you to the clinic?	Yes…………… 1<br>No …………… 2	If 2, go to Q # 28	
Q # 26	What is his / her job?	Office Job .... 1<br>Businessman ….2<br>Laborer ……….3<br>Unemployed … 4<br>Other ………... 0	If 4, go to Q # 28	
Q # 27	What is his/her monthly income?	Actual		
Q # 28	Because of cost, do you:			
	(a) Skip a pill or insulin shot?	Yes………………1<br>No ………………2		
	(b) Skip checking your blood sugar?	Yes………………1<br>No ………………2		
	(c) Skip consultation?	Yes………………1<br>No ………………2		
	(d) Other ___________________________	Yes………………1<br>No ………………2		
Q # 29	Who is responsible for the finances of your diabetes treatment?	Self …..…………..1<br>Spouse …………2<br>Parent ………..... 3<br>Brother/sister ….. 4<br>Son / Daughter ... 5<br>Employer ……… 6<br>Health insurance..7<br>Other ………..…0		
Q # 30	Any extra information on expenses incurred due to diabetes?	Actual		
